# A comparison among PCNL, Miniperc and Ultraminiperc for lower calyceal stones between 1 and 2 cm: a prospective, comparative, multicenter and randomised study

**DOI:** 10.1186/s12894-020-00636-z

**Published:** 2020-06-10

**Authors:** Giorgio Bozzini, Tahsin Batuhan Aydogan, Alexander Müller, Maria Chiara Sighinolfi, Umberto Besana, Alberto Calori, Berti Lorenzo, Alexander Govorov, Dmitry Y. Pushkar, Giovannalberto Pini, Antonio Luigi Pastore, Javier Romero-Otero, Bernardo Rocco, Carlo Buizza

**Affiliations:** 1 Department of Urology, ASST Valle Olona Busto Arsizio, Varese, Italy; 2 Department of Urology, University of Modena and Reggio Emilia, Modena, Italy; 3 Department of Urology, Spital Limmattal, Schlieren, Switzerland; 4 Department of Urology, A.I. Evdokimov Moscow State University of Medicine and Dentistry, Moscow, Russia; 5 Department of Urology, San Raffaele Turro Hospital, Milan, Italy; 6 Department of Urology, ICOT Latina, Latina, Italy; 7 Department of Urology, Hospital Universitario 12 de Octobre, Madrid, Spain

**Keywords:** PCNL, Percutaneous, Miniperc, Ultraminiperc, Lower calyces stones, Stone-free rate

## Abstract

**Background:**

Conventional Percutaneous Lithotripsy (PCNL) has been an effective, successful and easy approach for especially > 1 cm sized calyceal stones however risks of complications and nephron loss are inevitable. Our aim is to compare the efficacy and safety of PCNL, MiniPerc (MP) and UltraMiniPerc (UMP) for lower calyceal stones between 1 and 2 cm with a multicenter prospective randomized study.

**Methods:**

Between January 2015 and June 2018, 132 consecutive patients with single lower calyceal stone were enrolled. Patients were randomized in three groups; A: PCNL; B: MP; C: UMP. 44 patients for the Group A, 47 for Group B and 41 for Group C. Exclusion criterias were the presence of coagulation impairments, age of < 18 or > 75, presence of infection or serious comorbidities. Patients were controlled with computerized tomography scan after 3 months. A negative CT or an asymptomatic patient with stone fragments < 3 mm size were the criteria to assess the stone-free status. Patient characteristics, stone free rates (SFR) s, complications and re-treatment rates were analyzed.

**Results:**

The mean stone size were 16.38, 16.82 and 15.23 mm respectively in Group A, B and C(*p* = 0.34). The overall SFR was significantly higher in Group A (86.3%) and B (82.9%) as compared to Group C (78%)(*p* < 0.05). The re-treatment rate was significantly higher in Group C (12.1%) and complication rates was higher in Group A (13.6%) as compared to others(*p* < 0.05). The hospitalization was significantly shorter in Group C compared to Group A (*p* = 0.04).

**Conclusions:**

PCNL and MP showed higher efficacy than UMP to obtain a better SFR. Auxiliary and re-treatment rates were higher in UMP. On the other hand for such this kind of stones PCNL had more complications. Overall evaluation favors MP as a better indication in stones 1–2 cm size.

## Background

Lower calyceal (LC) stones approximately account for 35% of all renal stones [1]. Natural history of non-obstructing asymptomatic LC stone is not well defined, and the risk of progression is unclear. Although the question of whether calyceal stones should be treated is still unanswered and patients electing expectant management should be counseled regarding the potential for stone-related symptom progression and need for future intervention [2]. Stone growth, de novo obstruction, associated infection, stone composition, and acute and/or chronic pain are general indications for treatment decision. According to the European Association of Urology (EAU) guidelines as considered for LC stones, in case of unfavorable conditions for extracorporeal shockvawe lithotripsy (ESWL), endourological interventions such as Percutaneous Nephrolithotomy (PCNL) or Retrograde Intrarenal Surgery (RIRS) are recommended at first step [3].

LC stones have been a problem for both patients and urologists. Due to the anatomical variations of LC, especially considering for stone clearance, ESWL has been usually failed in here. Flexible ureterorenoscopic interventions have also gained popularity by time for especially endoscopic treatment of < 2 cm sized LC stones [3, 4]. But again because of some anatomical difficulties and durability of instrument it may not always be easy to perform RIRS for all LC stones [5]. Conventional PCNL has been an effective, successful and easy approach for LC stones however risks of complications and nephron loss are inevitable [2]. But regarded to the invasiveness and morbidities of PCNL, the optimal management of LC stones continue to be a dilemma that has recently received heightened attention among urologists. Moreover with time the miniaturization of PCNL also developed in order to diminish access related complications, bleeding and morbidities [6]. Conventionally the miniaturization of PCNL has defined by diameter less than 20 Fr [7]. The Minipercutaneous (MP)(16–18 Fr), Ultraminiperc (UMP)(11–14 Fr) and Micropercutaneous (MicroPNL) (< 10Fr) are currently feasible and modern alternatives with minimal invasiveness [2]. The MP is accepted as suitable for renal stones < 2 cm in size while UMP is for renal stones < 1.5 cm [7, 8]. A prospective multicenter randomized comparison among PCNL, MP and UMP for LC stones between 1 and 2 cm according to CT scan was performed to evaluate the efficacy and the safety of these procedures.

## Methods

Between January 2015 and June 2018, 132 consecutive patients presented with a singular 1–2 cm LC stone were enrolled in this prospective, comparative, multicenter and randomised study. The ethical committee approval was taken in 2014 thus gave the possibility to start at the same time in January 2015 for each participating center. Randomized number table was used for simple randomization. Patients were randomized into three groups; Group A: patients treated with PCNL; Group B: patients treated with MP; Group C: patients treated with UMP. The Hounsfield Unit (HU) of each group was 1234 ± 256, 1113 ± 341 and 1254 ± 289 for Group A, B and C respectively. The main inclusion criteria was related on stone’s maximum diameter in coronal images on CT scans. Exclusion criteria were the presence of coagulation impairments, age less than 18 or more than 75 (with increased risks of elongated procedure under general anesthesia leading with incomplete completion of interventions), presence of acute infection, presence of cardiovascular or pulmonary comorbidities. 8 patients for Group A, 9 for Group B and 7 for Group C were excluded. Patients were controlled with CT scan after 3 months. A negative CT or an asymptomatic patient with < 3 mm residual stone fragments and a negative urinary culture were the criteria to obtain the stone-free status (SFR). We accepted stone-free status as inferior to 3 mm and at least if the fragments we more than 2 in number with a residual area less than 9 mm^2^. A numerical pain scale from 0 to 10 was used for evaluation and comparison of pain levels. A statistical analysis was carried out to assess patients data, success and complications rates, re-treatment rate and need for auxiliary treatment. The informed written consent has been obtained from each participant, that study has been performed according to the Declaration of Helsinki, and the procedures have been approved by local ethics committee.

### PCNL, MP and UMP procedures

All PCNL procedures were started with the ureteral catheterization of stone side via cystoscopy. Under the semi-flex supine positioning access to kidney was performed by combined guidance of intraoperative ultrasonography (US) and fluoroscopy. After a successfully co-axial dilatation on guidewire up to a 30 CH Amplatz sheath, 800 μm Laser fiber with Litho 35 W (Quanta System, Samarate, Lombardia VA) was used for stone fragmentation (0.6–0.8 J 12 Hz) through a 24 Fr nephroscope (Karl-Storz) whereas retrieval grasper for stone extraction. A standart re-entry nephrostomy catheter was placed at the end and left for two days postoperatively.

The initial procedures before access, as cystoscopy and ureteral catheterization were similarly helded also in MP and UMP as during in PCNL. During MP, a 16 Fr nephroscope (Karl-Storz) was used with a smaller sheath size of 16.5/19.5 Fr (Karl-Storz) as compared to conventional PCNL. 550 μm Laser fiber with Cyber Ho was used by the help of a delicate forceps and nitinol basket for clearance of fragments. So at the end a small 10 Fr diameter nephrostomy catheter was placed.

Different than the two techniques, during UMP a gentle single step dilatation was needed for UMP sheath 11–13 Fr and Holmium laser energy was used through a 6 Fr nephroscope (Karl-Storz) for stone fragmentation. Again similar to MP, 550 μm Laser fiber with Cyber Ho was used. Neither graspers nor basket were used. Saline irrigation was mainly used to remove the stone fragments. Differently than other procedures, nephrostomy tube was not used.

For the all groups papilla access technique was used. The conventional PCNL could be used with balistic and ultrasonic lithotriptors as well but we used the laser fragmentation modality in order not to have bias regarding in between all three interventions. The energy settings did not differ in between procedures. Fragmentation method was used during PCNL whereas dusting method was used during MP and UMP. All the surgeons who had performed the procedures were all completed their learning curve on three techniques prior to this study with a minimum case record of 250 procedures for each technique. All the three procedures were done in two centers with six experienced surgeons.

### Statistical analyses

The Shapiro-Wilk normality test was used to evaluate the behavior of the continuous variables. The collected data were analysed by an online regression tool (Student’s t-test and chi-square test). The calculation of the sample size was done using “Creative Research System Software” indicating 95% as confidence level and a confidence interval (margin of error) of 15%. A post-hoc regression analysis was also performed on 3rd months SFR. For all statistical comparisons, significance was considered at *p* < 0.05.

## Results

Group A was consisted of 44 patients while Group B and Group C were consisted of 47 and 41 patients respectively. The mean stone size was 16.38 mm in Group A, 16.82 mm in Group B and 15.23 mm in Group C (*p* = 0.34). Our patient and stone related characteristics were all statistically similar in between the groups (*p* > 0.05) (Table 1). The overall SFR was 86.3% for Group A, 82.9% for Group B and 78.0% for Group C. The complication rates were 13.6, 4.2 and 2.4% respectively for group A, B and C. The re-treatment rates were significantly higher in group C compared to the other two groups (*p* = 0.007). Our analyze was by intention to treat so further on considering the re-treatment, RIRS was performed to get a stone free situation. The operating time did not show a significant difference in between the groups. The hospitalization was significantly shorter in Group C compared to Group A (*p* = 0.04) (Table 2). The post-hoc regression analysis on 3rd month SFR for groups was shown in Table 3. The complications of all groups were assessed and summarized as based up on clinical evidences (Table 4). The clinically evident complications were significantly higher in Group A as compared to the other groups (*p* < 0.05) (Table 4). The distribution of post-operative complications according to Clavien-Dindo was summarized in Table 5. The Clavien-Dindo scale of scores were higher in Group A as compared to the other groups (*p* < 0.05).

**Table 1:** The demographic and stone related characteristics of groups

	**Group A**	**Group B**	**Group C**	***p*value**
**Total patient (*n*** **= 132)**	44	47	41	0.44
**Age yrs** **(mean ± SD)**	53.3 ± 14.8	55.8 ± 16.1	54.8 ± 17.2	0.33
**Sex (M/F)**	23/21	20/27	22/19	0.23
**Height cm (mean ± SD)**	171.1 ± 2.5	175.8 ± 4.1	173.2 ± 2.3	0.40
**Weight Kg (mean ± SD)**	73.1 ± 4.6	77.9 ± 4.7	75.5 ± 7.1	0.66
**Stone side (Right/Left)**	25/19	22/25	21/20	0.52
**Stone size (mm)** **(mean ± SD)**	16.38 ± 2.9	16.82 ± 2.7	15.23 ± 3.3	0.34

Group A: PCNL, Group B: MP, Group C: UMP

**Table 2:** The comparison of re-treatment needs, complications and SFRs percentages of groups

	**Group A**	**Group B**	**Group C**	***p*value** **A-B**	***p*value A-C**	***p*value B-C**
**Need forre-treatment(%)**	6.8	4.2	12.1	0.17	0.41	**0.007**
**Operating time min ± SD**	45.9 ± 7.7	55.8 ± 11.4	59.3 ± 13.8	0.07	0.05	0.17
**Hospitalization days ± SD**	3.7 ± 1.5	2.7 ± 2.1	2.2 ± 2.1	0.06	**0.04**	0.18
**Complicationrate (%)**	13.6	4.2	2.4	**0.001**	**0.002**	0.25
**SFR at 3rd month (%)**	86.3	82.9	78	0.33	**0.022**	**0.02**

Group A: PCNL, Group B: MP, Group C: UMP, SFR: Stone-free rate. Re-treatment (Secondary Interventions): Retrograde Intrarenal Surgery (RIRS)

**Table 3:** Post-hoc logistic regression analysis on 3rd month SFR

	**SFR at 3rd month**		
**Group**	**Log-odds**	**Odds**	**Probability**
**A**	1.91	7.02	0.87
**B**	1.81	5.42	0.83
**C**	0.72	1.88	0.77

Group A: PCNL, Group B: MP, Group C: UMP, SFR: Stone-free rate

**Table 4:** The complications (Clinical evidences)

	**Group A (44)**	**Group B (47)**	**Group C (41)**	***p*value A-B**	***p*value A-C**	***p*value B-C**	**Total**
**UTI n (%)**	4 (9.2)	2 (4.2)	1 (2.4)	**0,0320**	**0,0230**	0,2300	7 (5.3)
**Gross haematuria** **n (%)**	2 (4.5)	0 (0)	0 (0)	**0,0020**	**0,0030**	10,000	2 (1.5)
**Severe painn (%)**	2 (4.5)	1 (2.1)	1 (2.4)	0,0500	0,0510	0,3475	4 (3.0)
**Totaln (%)**	8 (18.2)	3 (6.3)	2 (4.8)	**0,0410**	**0,0320**	0,1300	13 (9.8)

Group A: PCNL, Group B: MP, Group C: UMP, UTI: Urinary tract infection

**Table 5:** The postoperative complications (Clavien-Dindo classification)

	**Group A (44)**	**Group B (47)**	**Group C (41)**	***p*value** **A-B**	***p*value** **A-C**	***p*value** **B-C**	**Total**
**Overall complication** **n (%)**	8 (18.2)	3 (6.3)	2 (4.8)	**0,0410**	**0,0320**	0,1300	13 (9.8)
**Clavien score1–2** **n (%)**	6 (13.6) 4 UTI 2 Severe Pain	3 (6.3) 2 UTI 1 Severe Pain	2 (4.8) 1 UTI 1 Severe Pain	**0,0280**	**0,0190**	0,0961	11 (8.3)
**3a-5n (%)**	2 (4.5) Gross Hematuria	0 (0)	0 (0)	**0,0020**	**0,0030**	10,000	2 (1.5)

Group A: PCNL, Group B: MP, Group C: UMP, UTI: Urinary tract infection

## Discussion

The LC stones have always been problematic in order to obtain a total stone clearance. It is very well discussed in literature as a systematic review and meta-analysis showed PCNL and RIRS were superior to ESWL in stone clearance and even PCNL is more effective then RIRS [9]. In 2017 Bozzini et al. performed a prospective randomized comparison among ESWL, PCNL and RIRS on < 2 cm sized LC stones [10]. ESWL was failed against PCNL and RIRS considering SFR and re-treatment rates(*p* < 0.05). The SFRs were significantly higher in PCNL (87.3%) and RIRS (82.1%) as compared to ESWL (61.8%). The duration of procedure and hospital stay, radiation exposure favored RIRS against PCNL [10]. RIRS looks a feasible and better option against PCNL when complication rates were also taken in consideration. But still RIRS may not be performed on all LC stones successfully due to anatomic variations as narrow ureter or calyceal infindibulum. So among with the miniaturized PCNL modalities as MP, UMP and MicroPNL there need of a decision to choice the priority for most effective and safe intervention against < 2 cm LC stones even in case of narrow calyx existence [2].

The MP was first described and experienced with a single step dilatation with usage of 11 Fr sheath on 11 cases at preschool age [11]. At the same year an initial results from an adult study appeared [12]. Nine patients with < 2 cm stones were treated with 13 Fr MP. 89% stone-free rate was achieved while with better outcomes in selected patients favoring MP for blood loss, hospital stay and postoperative pain [12]. Despite this, Giusti et al. declared that MP had no obvious advantage against PCNL and concluded as tubeless PCNL could be a better option [13]. But Knoll et al. defended that not all tubeless PCNL procedures has ended with delighted results. Some cases need auxiliary interventions. Even being a limitation in their study that stone sizes were smaller in MP patients; the results were comparable in means of safety and effectiveness. More, the short hospital stay, less pain and more possibility for tubeless completion of operations has favored MP [14]. The MP studies have showed comparable and equal SFR and reduced comorbidities compared to conventional PCNL [8, 14, 15]. ElSheemy et al. compared MP and standard PCNL through 18 and 30 Fr tracts respectively [16]. According to their results there was significant difference on SFR in between patients who had larger stone burden (>2cm^2^). But for those with singular stones or stone burden ≤2 cm^2^; there was no significant difference in SFR and also MP showed lower complications compared to standard PCNL [16]. Similar to the current literature, in our study there was no significant difference in between SFR of PCNL and MP(*p* = 0.33) while a significant difference in between complication rates (*p* = 0.001) (Table 2).

In 2013, the UMP technique was published with initial experiences in literature [17]. Desai et al. used a 6 Fr mini nephroscope through a 11/13 Fr metal sheath on 36 consecutive patients with stone size of < 2 cm [17]. Similar to our access method, ultrasonographic guidance was also used in addition to fluoroscopy. Not all stones were located in LC. The LC stone ratio and LC access ratio were 27.8 and 38.9% respectively while 8.3% of their patients had multiple calyceal stones. Their immediate (postoperative 1st day) and total (postoperative 1st month) SFR’s were 88.9 and 97.2% respectively [17]. More, their significant complication (urosepsis, extravasation and fever) ratio was 16.7% and need for a second intervention was 2.8% whereas ours were 2.4 and 12.1% respectively. This inverse ratio may be explained with the differences between operative time, stone analysis, surgeon experience or other patient characteristics in between the studies.

In a cohort study of 98 consecutive UMP patients, the mean stone size was 15.85 ± 4.53 mm which was comparable of ours (15.23 ± 3.3 mm) but their postoperative SFR on 1st month control was 83% [18]. Jones et al. published a systematic review study investigating the role of MicroPNL and UMP. Across seven studies a total of 262 patients were undergone UMP with a mean of stone size 18.6 mm, SFR of 88.3% and complication rate of 6.2% [19]. In our study, we calculated the SFR on 3rd month control. The SFR of our UMP was calculated as 78% which was also significantly lower than our PCNL and MP groups.

Ganpula et al. discussed well the differences between PCNL, MP and UMP [7]. The cross-sectional area and length differences of access sheats and more the smaller fragments obtained during MP all create a superior fragment vacuum clearance during MP compared to PCNL. Also sheath sizes of MP may give a chance to use flexible nephroscope for stone fragments in smaller different calyces [7]. So these all may participate to the similar SFR in between PCNL and MP. Again in another study, during the interventions on 15–30 mm renal stones, 16.5 Fr MP showed comparable SFR but lower complications as compared with 24 Fr PCNL [20]. According to a recent review study the terminology seems to be confusing in between the modalities but all miniaturized tract size interventions result with better outcomes in terms of pain and complication rates whereas comparable SFR with standart PCNL [21]. Depending on our comparison in between MP and UMP complication ratios were comparable but our SFR results in between MP and UMP were statistically significant(*p* = 0.02). According to our results mean stone size were similar as 16.38 mm, 16.82 mm and 15.23 mm respectively. The UMP looks like an alternative to MP but may not be satisfactory especially for interventions on stones with > 1.5 cm sized. The UMP may also be a good alternative to RIRS especially for medium sized (< 1.5 cm) LC stones. This mismatch between the ideal indication for UMP and the stone size treated with UMP in this study may have meaningful bias on the results and downgraded the potential efficacy of UMP. However, currently due to the lack of literature there are no clear guideline on MP and UMP.

The PCNL was at highest risk of complication co-existence. The clinically evident complications in Table 4 were all higher in PCNL compared to MP and UMP. Only among those, between PCNL and MP the severe pain seems to be questionable(*p* = 0.05). According to Mishra et al. the need of postoperative analgesics were similar between PCNL and MP [8]. More in contrast to PCNL, none of the patients who were undergone MP and UMP procedures needed a further surgical or endoscopic interventions according to Clavien-Dindo classification [22] score of >III (Table 5). Also there was no difference among clinically evident complications between MP and UMP. Careful selection of patients, experience of surgeon and special equipment for each intervention is essential.

The number of the patients in each groups may be a statistical limitation. So we can assume that surely the tendency (obtained by univariate analysis) would be better confirmed by randomized control trial with higher numbers on which we are already working.

Here in our study a limitation may be lack of evaluation according to the stone analysis or Hounsfield unit (HU). We did not include pediatric age group (< 18) in order not to increase the heterogeneity with extra choice of MP and UMP. Moreover, exclusion of patients more than 75 years old seems to be a potential selection bias but but increased risks with older age under general anesthesia may require incomplete termination of procedure which may also be a cause of bias on postoperative outcomes.

Tubeless or non-tubeless comparison may be also considered in future studies. MP may seem to be supported as first choice of PCNL subtype considering the 1–2 cm sized LC stones. UMP may be recommended for certain selected patients. Further studies with giant patient groups may show comparable SFR in between MP and UMP. As last, the use of the same Holmium laser energy during the interventions might give the possibility to focus only on the technique chosen and not a different kind of energy to break the stone.

## Conclusion

PCNL and MP showed higher efficacy than UMP in order to obtain a better SFR. Auxiliary and re-treatment rates were similar between PCNL and MP but differed in between MP and UMP. On the other hand for such this kind of stones PCNL had more complications among all. The MP and UMP find a better indication whereas MP seems to be the preferential alternative for 1–2 cm LC stone intervention among the others. Further randomized comparison between RIRS and MP in this setting is needed.

## Data Availability

The datasets used and/or analysed during the current study are available from the corresponding author on reasonable request.

## Abbreviations

PCNL: Percutaneous Lithotripsy
MP: Minipercutaneous lithotripsy
UMP: Ultraminiperc lithotripsy
ESWL: Extraschock vawe lithotripsy
RIRS: Retrograde intrarenal surgery
SFR: Stone-free rate
LC: Lower calyceal
EAU: European Association of Urology
